# DIdM-EIoTD: Distributed Identity Management for Edge Internet of Things (IoT) Devices

**DOI:** 10.3390/s23084046

**Published:** 2023-04-17

**Authors:** Kazi Masum Sadique, Rahim Rahmani, Paul Johannesson

**Affiliations:** Department of Computer and Systems Sciences, Stockholm University, SE-164 07 Kista, Sweden; rahim@dsv.su.se (R.R.); pajo@dsv.su.se (P.J.)

**Keywords:** Distributed Ledger Technology (DLT), blockchain, Internet of Things (IoT), identity management, identity authentication, authorization, security, trust, privacy, scalability

## Abstract

The Internet of Things (IoT) paradigm aims to enhance human society and living standards with the vast deployment of smart and autonomous devices, which requires seamless collaboration. The number of connected devices increases daily, introducing identity management requirements for edge IoT devices. Due to IoT devices’ heterogeneity and resource-constrained configuration, traditional identity management systems are not feasible. As a result, identity management for IoT devices is still an open issue. Distributed Ledger Technology (DLT) and blockchain-based security solutions are becoming popular in different application domains. This paper presents a novel DLT-based distributed identity management architecture for edge IoT devices. The model can be adapted with any IoT solution for secure and trustworthy communication between devices. We have comprehensively reviewed popular consensus mechanisms used in DLT implementations and their connection to IoT research, specifically identity management for Edge IoT devices. Our proposed location-based identity management model is generic, distributed, and decentralized. The proposed model is verified using the Scyther formal verification tool for security performance measurement. SPIN model checker is employed for different state verification of our proposed model. The open-source simulation tool FobSim is used for fog and edge/user layer DTL deployment performance analysis. The results and discussion section represents how our proposed decentralized identity management solution should enhance user data privacy and secure and trustworthy communication in IoT.

## 1. Introduction

The research and development of connected devices and services are increasing every day. Professor Ashton introduced the phrase Internet of Things (IoT) in 1999 [1]. An IoT news forecast mentioned that by 2030, the number of connected devices worldwide would reach 24.1 billion [2]. The internet-connected devices and services improve our lifestyles by increasing work efficiency and productivity with innovative IoT applications. The benefits of the IoT are no longer limited to urban areas, also improving the quality of life and work in rural areas. The most common use cases are smart grid, smart city, smart home, smart healthcare, [3] etc. All these innovative IoT applications are implemented in the physical world, where individuals’ and organizations’ data are transferred via the internet [4]. As the innovation of IoT applications is currently an ongoing process, the need for trustworthy and secure communication increases. Identity management and authentication are essential for trustworthiness and secure communication [5]. In the nascent stages of the Internet, IPv4 was deployed to manage the identification of the increasing number of devices. IPv4- and IPv6-based device identification are still in use for communication between devices [6]. To ensure small range communication, Bluetooth, RFID, and NFC were invented [7]. Cloud computing is an emerging technology. Though there are several benefits of all these currently available identity and communication solutions, these are still not fully compatible with the future needs of trustworthy and secure communication for the IoT paradigm [8]. The IoT device and services are heterogeneous, and they need real-time decision-making within a short time period [9]. IoT devices are resource-constrained and designed to perform specific tasks in the deployed environment [10,11]. Almost all the currently deployed IoT solutions are centralized, and the cloud layer is the main place for device identification and authentication. In these scenarios, IoT devices are controlled and authenticated from centralized servers [12]. However, centralized solutions are not very feasible for an IoT solution where real-time decision-making is required [13]. Additionally, most IoT devices are deployed in human society. The intruders can collect confidential data from IoT devices, gateways, during transactions to the cloud server, or even at the cloud servers due to lack of security, authentication, and authorization [14].

Moreover, IoT devices need to trust and collaborate at the edge layer without obtaining any identity verification from the cloud layer [13,15]. DLT and Blockchain-based security solutions are growing in popularity as they provide secure and trustworthy data storage and management [16]. The first use case of Blockchain was Bitcoin, introduced by Nakamoto in the year 2009, where he presented Bitcoin as a digital currency [17]. After the first innovation of Blockchain, different business and communication sectors are adopting the concept of Blockchain in their current use cases [18,19]. As a result, many types of DLT have been introduced. Many recent researchers addressed the Blockchain-based trust, identity, and security solutions for IoT. One of the main advantages of DLT is decentralization. Our previous work proposed a distributed and decentralized architecture for identity management and secure communication for edge IoT devices [20]. During further research, we found that adopting DLT with our proposed model could be the best fit for the increasing demand for secure communication and trustworthy identity management for edge IoT devices. Motivated by the above information, we propose a DLT-based identity management and secure communication solution for the IoT paradigm which is distributed and decentralized. We have performed a detailed literature review in our research domain, compared our proposed solution with the recently proposed IoT device identity management, and described how our proposed model would improve security, trust, and privacy for IoT solutions. We have also performed formal and informal verification of the proposed concept.

Below, we have listed our contributions in this article:

- In the Introduction section, we present an overview of the need for a distributed identity management system for security, trust, and privacy at edge IoT devices;
- A comprehensive survey on the DLT and related terms is performed, and the results are presented in the Background and Motivation section;
- We have also studied and summarized different DLT used for identity management within the IoT domain;
- We have proposed a new DLT-based distributed identity management model for IoT which will enhance data security, trust, and privacy;
- Additionally, we have presented the evaluation of our proposed architecture using SPIN, Scyther and FobSim simulators.

The rest of the paper is organized as follows:

- In Section 2, we present a detailed review of the DLT followed by our motivation to use DLT-based identity management for security, trust, and privacy at the edge IoT devices;
- Section 3 examines the related works, where recent results on DLT for identity management are discussed;
- Section 4 presents our proposed DLT-based identity management model for security, trust, and privacy at edge IoT devices;
- In Section 5, we present the performance measurement results of the simulation;
- In Section 6, we discuss different common qualities of an identity management system;
- Section 7 presents the conclusion of our work, and this paper ends with a future work description.

The following section describes our research background and a comparative presentation of DLT solutions and their connections with the IoT domain.

## 2. Background and Motivation

As discussed above, the use of Blockchain in different sectors is increasing on a daily basis due to the decentralized and self-adaptive approach of DLT. The use case of DLT and Blockchain in the IoT domain is also vast. Blockchain itself is a specific type of Data Structure (DS), which is implemented in many of the distributed ledgers. Though Blockchain and DLT are correlated, not every DLT needs to use blockchain technology [21]. DLT can be categorized based on different criteria. Selection of any specific DLT solution for any use case is difficult due to different deployment environments. For example, some use cases require public access to data, and others need private access. Below, we present the definition of DLT, followed by a categorization of DLT and how they are used in the IoT paradigm.

### 2.1. Distributed Ledger Technology (DLT)

**Figure 1:**
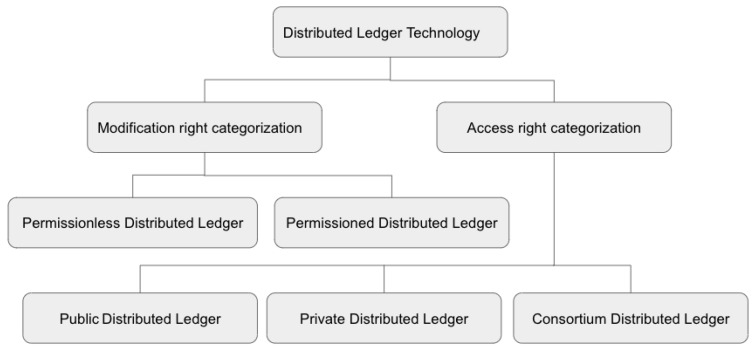
Categorization of DLT based on access and modification rights.

A distributed ledger is a specific type of register that is shared and maintained by different entities distributed and connected over the Internet [21]. DLT allows data sharing between different entities without any third party or central authority to verify the transitions performed by various entities. Additionally, DLT allows distributed and replicated storage capability, which allows data verification and validation [22]. The five main features of any DLT are: (1) data storage over distributed networks and sharing the identical copy on multiple storage, (2) validation of data independently by different participants, (3) agreement on the transaction by multiple parties (4) validation of changes in data by any participant of the network, and (5) hard to change the values in a transaction, namely temper resistance [22]. As presented in Figure 1, based on the modification right, distributed ledgers can be categories as: (1) Permissionless Distributed Ledger and (2) Permissioned Distributed Ledger. Based on the access right, distributed ledgers can be categories as: (1) Public Distributed Ledger, (2) Private Distributed Ledger and (3) Consortium Distributed Ledger.

#### 2.1.1. Permissionless Distributed Ledger

A distributed ledger where any network entity can modify the entry is called a permissionless distributed ledger [23]. It is a shared ledger between all the network entities, and no restrictions or pre-registration requirement are applied. An example of a permissionless distributed ledger is Bitcoin [23]. A permissionless ledger could be more effective where multiple parties create entries to the ledger without any previous access permission documented.

#### 2.1.2. Permissioned Distributed Ledger

A permissioned distributed ledger only allows modification by a group of entities based on the context it is implemented [23]. A modifier needs to be registered and has the right to perform any modification. An example of a permissioned distributed ledger is Hyperledger [23].

#### 2.1.3. Public Distributed Ledger

As per the name, a public distributed ledger is publicly available, and there is no restriction on the network access of its entity [23]. The use case of the public distributed ledger could be a public Wi-Fi network that is publicly available for anybody to connect their device and use the internet service.

#### 2.1.4. Private Distributed Ledger

A private distributed ledger is implemented and published for use within a specific group of entities based on its context [23]. For example, it could be people within an organization or a set of Wi-Fi networks or devices with a specific range of IP addresses and not publicly published.

#### 2.1.5. Consortium Distributed Ledger

A consortium-based distributed ledger or consortium blockchain is a hybrid implementation based on public, and private ledger, in which different authorities can add entries based on their access right, and can access the data based on their access right defined based on their role and permissions set up in the system [24,25,26,27]. An example implementation of consortium-based DLT is an inter-organizational ledger where multiple parties can add entries and view the entries [28].

The use of the type of DLT depends on the context in which it is deployed. For example, a multinational organization may need to have private DLT for communication between the offices in different cities and countries. However, the same organization may also need consortium DLT for interaction with their customers and partners, and public DLT for managing leads from prospective customers. A similar concept is applicable for the use of DLT in the IoT domain. Not all types of DLT are suitable for the IoT paradigm. Although the initial use case of blockchains and distributed ledgers was to allow public access to the data and to save the history of financial transactions, its use case is much broader today. Almost every multidisciplinary area implements DLT, where computer science can have a partial footprint. For example, law and order, a chain of custody for digital forensics, smart healthcare, and so on. The use case we have covered in this paper is identities for everything: users, user’s devices, and end IoT devices without compromising the user data privacy. Below, we present the definition of blockchain.

### 2.2. Blockchain

Blockchain is a type of DLT. Blockchain uses a cryptographic hash function to keep the integrity of each block [21]. Each of the new blocks added to the chain holds its hash and a hash of the previous block, which introduces the immutability of any change in the data at any block changing its respective hash. As a result, a change is easily detected consequently invalidating the content of the block [29]. Another criterion of Blockchain is P2P communication, where no central authority is involved, and all the nodes in a blockchain network hold the same copy of the data [30]. In case any change is required, it needs to be changed in all nodes through the network, so a new entry is added instead of rewriting the old record [31]. As the nodes within a blockchain network hold identical copies of the data, it ensures consistency and transparency within the specific blockchain network [32].

The heart of Blockchain and DLT is consensus mechanisms. Different types of consensus mechanisms are applied in different distributed ledger technologies. The consensus mechanisms have benefits and downfalls. When it comes to the IoT domain, not every consensus mechanism applies to resource constraint IoT devices [2]. Below, we present the definition of consensus mechanism followed by a description of different types of consensus mechanisms and their use in the IoT domain.

### 2.3. Consensus Mechanisms

**Figure 2:**
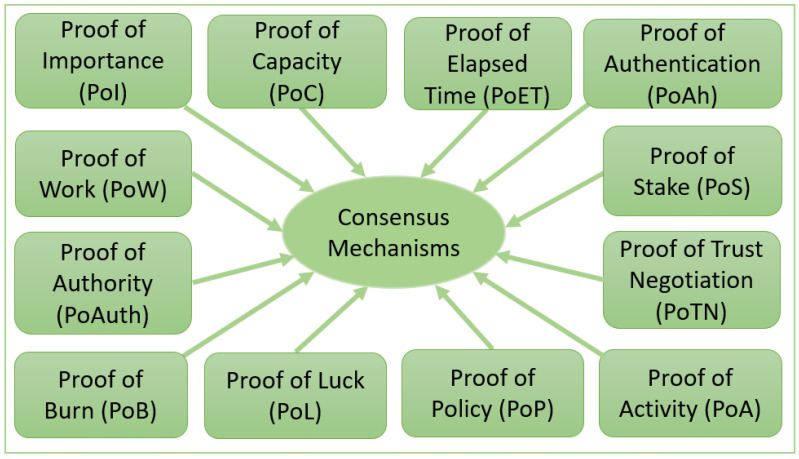
Representation of popular proof-based consensus mechanisms.

In an IoT paradigm, decision-making is an everyday use case while receiving and processing data from trusted and untrusted data sources. Identifying the trusted entity within a public internet network is also a problem. Consensus algorithms are used widely in different applications to make decisions, select the leader, and synchronize the state between distributed systems [33]. Consensus mechanisms relate to different types of proofs. Below, we discuss a few types of proofs used in distributed ledger technologies and Blockchains. We have explored these proofs considering their relationship to the IoT paradigm. Figure 2 represents 12 popular proof-based consensus mechanisms that are discussed in detail below.

#### 2.3.1. Proof of Work (PoW)

Proof of Work (PoW) is a miner-based consensus mechanism where network entities solve complex mathematical problems to add the following block in a chain of blocks. It is also called a leader election protocol because the winning miner achieves an award to be the leader for solving tricky computational puzzles [34,35,36,37,38]. To solve the puzzles, PoW requires high computational energy. PoW is suitable for public distributed ledger [36] as it is permissionless DLT [39,40]. As IoT devices are resource constraints, the PoW consensus mechanism cannot be used. The authors in [34] proposed a modified version of PoW called Green-PoW. They claimed that the algorithm required 50% computational energy, but it is still not a suitable solution for Fog and Edge-based DLT solutions. Furthermore, the distributed identity management solution for IoT required a combination of private and consortium-based distributed ledger solutions, which cannot be achieved with a high-load PoW consensus protocol.

#### 2.3.2. Proof of Capacity (PoC)

Proof of Capacity (PoC) is another miner-based consensus mechanism where the miners use their hard disk space to gain the chance to mine the next block [35,36]. As IoT devices and IoT infrastructure built at the edge layer are not much resource-heavy, proof of capacity is not a suitable consensus mechanism for distributed ledgers used for any IoT solutions. Furthermore, PoC is for a public distributed ledger [36,40] solution and is another permissionless DLT [39,40], so it is not of use in achieving the goal for our proposed model.

#### 2.3.3. Proof of Elapsed Time (PoET)

Proof of Elapsed Time (PoET) is another type of verification mechanism which can be used for permissioned Blockchain. It has low power consumption and latency compared to PoW [35,36]. In PoET, a leader is selected based on a random lottery on a first come, first serve basis, which means the miners do not need high computation power to solve puzzles [35]. PoET is one of the consensus mechanisms where a Trusted Execution Environment (TEE) is used for waiting time and equal distribution of mining opportunities [38]. PoET can be deployed both in private [40] and public distributed ledger [36] and it supports both permissionless and permissioned ledger [40]. It is one of the consensus algorithms which could be suitable for edge identity management modules.

#### 2.3.4. Proof of Stake (PoS)

Proof of Stake (PoS) is another consensus protocol that does not require high computational resources for solving puzzles such as the PoW [37]. In PoS, a leader is also selected by lottery. The amount of stake increases the possibility of being selected in the lottery [35]. PoS is one of the lightweight consensus protocols. PoS is categorized as Delegated Proof of Stake (DPoS) [36,38] and Leased Proof of Stake (LPoS) [36,38]. These consensus algorithms are also unsuitable for our proposed edge IoT solutions because there is no cryptocurrency involved in our discussed IoT solution. Furthermore, PoS is suitable for public distributed ledger [36,40] and a permissionless DLT [39], so it is not ideal for our proposed model.

#### 2.3.5. Proof of Importance (PoI)

Proof of Importance (PoI) is another type of PoS consensus protocol where the network activities of a node are considered a plus point for becoming the leader [35,36,37,38]. PoI can be used both in the public and private distributed ledgers [36]. It is also a lightweight protocol that requires fewer computational resources, and the importance is calculated based on the interaction of the wallet and the amount of currency the node owns [37]. Though it is a lightweight consensus mechanism, it is more suitable for cryptocurrency and not suitable for the IoT domain.

#### 2.3.6. Proof of Activity (PoA)

Proof of Activity (PoA) is a combination of PoW and PoS [35,37,38]. A miner needs to solve cryptographic puzzles as PoW, and it also needs to have a significant share of coins in the network. PoA is designed for public DLT and is a permissionless DLT [39,40], but in our proposed model, we suggest a solution based on private and consortium distributed ledger as it is not suitable for our proposed model. This protocol requires high computational power as it is a PoW-based protocol, so it is unsuitable for fog-edge layer deployment and edge IoT solutions [38].

#### 2.3.7. Proof of Burn (PoB)

Proof of Burn (PoB) is another extended version of PoW and PoS consensus protocols. Here, the miners burn coins to obtain their turn for mining [35,36]. PoB is a permissionless DLT [39,40] and suitable for public [40] deployment. Furthermore, this consensus protocol requires more computational power, which is inappropriate for edge-fog layer IoT solutions [38].

#### 2.3.8. Proof of Luck (PoL)

In Proof of Luck (PoL), a leader is selected for mining by random number generation in Trusted Execution Environment (TEE). It is another lightweight protocol requiring less computational power [37,38]. This consensus mechanism requires a special type of hardware for TEE [38].

#### 2.3.9. Proof of Policy (PoP)

Proof of Policy (PoP) is a consensus protocol that is also lightweight. It is another PoS based protocol where the attribute-based ring signature is used to determine the miner. It requires fewer computational resources than PoW and PoS. PoP is suitable for deployment in a private DLT environment [41].

#### 2.3.10. Proof of Trust Negotiation (PoTN)

Proof of Trust Negotiation (PoTN) is a trust-based protocol where only trusted miners are selected for adding blocks. Here, random miners are determined based on their trust level [37]. It is one of the protocols that can be used in permissioned DLT deployment with a private ledger because here, miners can be elected based on their trust negotiation capabilities.

#### 2.3.11. Proof of Authority (PoAuth)

Proof of Authority (PoAuth) is a modified form of PoS [35]. In PoAuth, miners receive opportunities based on their identity. It is one of the lightweight consensus mechanisms suitable for permissioned DLT [38]. This consensus protocol could be ideal for our proposed model, but Proof of Authentication (PoAh) is more suitable for our scenario, and it is described below.

#### 2.3.12. Proof of Authentication (PoAh)

Proof of Authentication (PoAh) is one of the consensus mechanisms that requires very limited computational resources and low latency [37,42,43,44,45,46,47,48]. In PoAh, a node can be selected for adding a block to the chain based on authentication status. PoAh can be deployed in private DLT [42,44,45] and is suitable for a permissioned distributed ledger. PoAh is also ideal for integration with fog and edge computing [46]. Recent research identified PoAh as one of the consensus algorithms suitable for edge IoT solutions [42,43,45,47,48]. We have also selected PoAh to demonstrate our proposed model.

Many other consensus algorithms are not described in detail as those are not much relevant but can be found in Table 1. From the above discussion and categorizations, there is no single way to achieve consensus in DLT. Many types of distributed ledgers are introduced based on different consensus mechanisms.

### 2.4. Summary

The use of DLT, specifically blockchain-based consensus algorithms, became de facto in the domain of digital currencies after the first proposal of Bitcoin. Though the adaptation of Blockchain in IoT is ongoing research, PoW and the miner-based consensus are not very suitable for the edge IoT devices as those are resource constraint devices and are not suitable for resource-heavy calculations. Motivated by this, we have performed our research within the domain and proposed a new hybrid distributed ledger-based approach for identity management of IoT devices which will enhance security and trust at the edge layer of IoT paradigm. Before describing our proposed solutions, we present an extensive literature review on the state-of-the-art identity management for IoT devices, which are related to our research.

## 3. Related Works

In this section, we have presented a comprehensive survey on related works. Many recent papers have discussed DLT especially Blockchain-based identity management for IoT devices [49,50,51,52,53,54,55,56,57,58,59,60,61,62,63,64,65,66,67,68]. However, some of them also discussed the combination of machine learning and other technologies with the blockchain-based solution and some discussed the use of IoTA for a similar purpose. We briefly present a review of related works below.

In [49], a detailed survey on blockchain-based identity management for the IoT was presented. The authors discussed scalability, interoperability, and mobility as essential requirements for the design of identity management systems for the IoT. The authors also presented the drawbacks of centralized identity management solutions. Furthermore, the authors described traditional identity management solutions, followed by different blockchain-based industrial solutions and currently proposed identity management solutions by academics. Finally, the authors discussed different challenges related to identity management, access controls, trust management, privacy, and performance evaluation. In an earlier survey [50], the same authors presented comparative presentations on different initiatives on identity management, as well as comparisons between different blockchain-based identity management solutions. Another survey on Blockchain for identity management presented a detailed analysis of six use cases where blockchain is used for identity management. The discussed solutions are Namecoin, uPort, Sovrin, Blockstack, ShoCard and Jolocom [51].

In [52], the authors suggested a lightweight blockchain-based authentication and authorization solution framework for healthcare IoT systems. The proposed solution used the probabilistic model. In the proposed model, the authors recommended a miner-based solution and the authentication and authorization process take place at the cloud layer. Though the authors used a fog layer in their recommended model, the Blockchain solution is integrated with the cloud layer. Moreover, the authors recommended the solution based on the PoW consensus mechanism, which is a heavyweight solution and not very realistic for edge-based IoT scenarios. This type of solution can be applied for a model where most of the operations, decision-making, and data transfers involve the cloud layer.

In [53], the authors proposed a consortium-based centralized identity management solution for the IoT. They discussed certificate management for the IoT entities and presented the architecture. Furthermore, they implemented their recommended model in a laboratory environment with Hyperledger Fabric. This solution is suitable for cloud-based and centralized solutions for IoT device identity management. In another publication [54], the same group of authors presented a combined solution for IoT access control using capability-based access control and consortium-based blockchain technology. In our research, we focus on edge-based identity solutions, which will improve the authentication and authorization process at the edge layer.

The authors, in [55], recommended a model with device clustering and blockchain for the authentication of devices. They also suggested the solution with local blockchain and global blockchain layer for local authentication and authorization process, which shares a slight similarity in architecture with our recommended model, but we have developed our model based on our previous works [5,20], where we also designed and verified secure communication protocol for IoT devices and their work is based on device clustering. Moreover, we propose the use of private ledger at the edge layer (local blockchain layer) to make it secure within a certain location and consortium ledger at the cloud layer (global blockchain layer) where inter-organizational collaboration could be required for sharing of identity information.

In [56], the author recommended a role and permission-based decentralized model for access control of IoT. Wireless sensor nodes connect with the blockchain system via the management hub and miner networks for performing smart contracts. The managers assign access permissions to the IoT nodes. As we have already described above, a miner-based solution is not suitable for edge-based IoT solutions.

In [57], the authors suggested a cross-domain secure authentication model for the industrial IoT devices. The model used layered architecture and localization of IoT devices by separating in different domains, which we think is a good concept, and we also consider similar concepts in this paper as well as in our previous works [5,20]. However, in their recommended model they proposed a central server for each domain, which creates the possibility of a single point of failure, on the other hand, we propose identity management using DLT at the edge layer of the IoT paradigm.

In [58], the authors presented identity management for cloud-based IoT applications based on Blockchain. The proposed use case uses facial recognition of students to identify them for the printing service at any institute. They recommend the use of cloud services for the user data for their blockchain-based solution. This is useful in that certain context, but it is not applicable for distributed IoT end devices and a generic model for identity management.

User access control to IoT devices using Blockchain technology has been presented in many recent publications [59,60,61,62,63,64,65,66]. A user access control based on ciphertext-policy attribute-based authentication (CP-ABE) and IoTA is recommended in [59], though the paper title is about IoT, the focus is more towards token-based authorization of user’s handheld devices for accessing IoT devices. Another work recommended user access control and authorization to IoT resources for its end users in a smart city environment. The proposed solution was implemented in the private Ethereum blockchain [60]. The authors also presented the smart contract, and blockchain interaction with the application. Smart-contract-based access control for edge computing is suggested in another research publication [61]. Here, the authors use Colored Petri Net (CPN) to present their blockchain-based edge computing solution. An Ethereum blockchain and smart contracts-based identity management solution was also proposed in [62], where device ownership is mapped with the device using Blockchain. A decentralized identifier and blockchain-based user-centric IoT device identity management solution is recommended in [63], where the focus is also IoT device ownership using blockchain technology. Another paper presented a private distributed ledger based IoT identity management solution for enterprise users [64]. In [65], user identity management and device ownership using Blockchain, attribute management via certificate authority is recommended. Though the proposed identity solution is blockchain based, it still has a dependency on certificate authority which is not fully a distributed solution. Furthermore, a blockchain- and capability-based IoT user access control model is recommended in [66].

A security model with an identity and authentication framework is recommended in [67]. The authors here presented the security challenges in IoT and the requirement of identity management for IoT devices. The recommended solution is namely BCoT sentry, where device authentication is carried out at the Blockchain gateways. They also implemented a prototype in Ubuntu virtual machine to demonstrate their proposal. This approach is good, but their implementation is different from ours, as we have presented the localization of the problem at the edge or fog layer of IoT to improve the IoT security at the lower layer of the IoT paradigm.

In [68], the authors presented a blockchain and Unique-ID-based decentralized solution model for IoT devices. This solution also has some similarities with our model, but in our model, we have recommended a solution that is distributed and decentralized but also uses scalability feature of cloud layer of IoT paradigm.

As we found from the above literature review, a generic model for identity management of edge IoT devices is still an open issue. Many of the works are more focused on user access control to the IoT devices. As a result, in our recommended model, we have presented a generic solution for distributed identity management for the IoT device itself which can be adapted to different use cases, for example, smart city, industrial IoT, smart home, smart agriculture, etc. The below section presents the architecture and details of our proposed distributed ledger-based identity management solution for edge IoT devices.

## 4. Our Approach

In this section, we describe our proposed system. First, we presented abbreviations and descriptions of different terms mentioned within our proposed model. After that, we clarify different assumptions we have considered, followed by the offered system model description, workflow of the system, and smart contracts and few algorithms for supporting the proposed model.

### 4.1. Notations of Terms

We have considered different terms throughout the paper. Table A1 in the appendix section presents abbreviations and descriptions of different notations of terms.

### 4.2. Assumptions

The proposed identity management architecture with related protocols is valid with the following assumptions. The assumptions support the further implementations of the proposed model.

- Our proposed decentralized model has restricted access rights to identity information within a particular area. Access to the identity information for devices within other blockchain networks can also be possible via the global cloud layer blockchain network;
- Any device collaborating with any other device via the Blockchain-based identity management nodes must be initially registered to the network, either manually or via a mobile operator;
- A valid national identity number and photo Id are required for user registration, which should be stored in Blockchain;
- After the initial registration, the users and devices become part of a local blockchain network where it is located;
- Data communications between devices, gateways, and servers are carried out using a secure communication protocol that was proposed in our previous work;
- The symmetric keys for secure communication between IoT devices and edge gateways are transferred securely using the secure communication protocol proposed in our previous work.

### 4.3. System Model

As we have mentioned earlier, in our previous work, we described a decentralized identity management model for edge IoT devices [20]. We also proposed a layered architecture for security in IoT [69]. In the current paper, we combined those models with DLT and smart contacts. Our proposed model is based on the concept of shared ledger [70], which can also be a distributed ledger but a private permissioned distributed ledger or a private consortium ledger where the entries are not shared publicly but only between the nodes where mutual trust exists. Furthermore, they are located within a particular location. The region for proposing a hybrid architecture with a combination of mixed access rights is to protect identity information from a broader range of users, which reduces security risks and increases privacy. The private permissioned DL should store IoT device identity information at the fog layer within a Local Identity Provider (LIdP), which could be within a specific location, organizational network, or service provider. The consortium ledger is used for inter-organizational collaboration and identity data transfer in case it is required at the cloud layer within a Global Identity Provider (GIdP), which will allow interoperability and mobility of the IoT devices.

**Figure 3:**
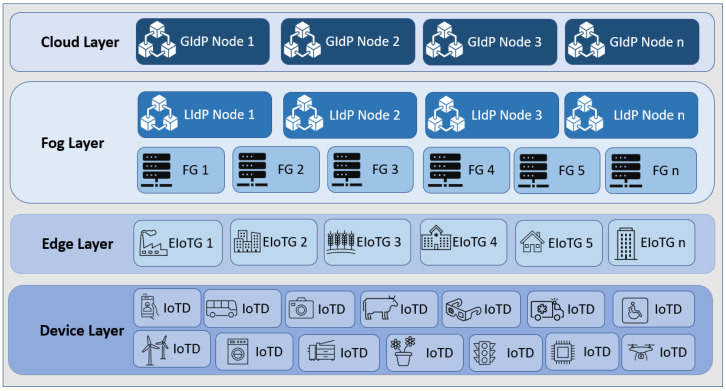
Overall architecture of the proposed model.

**Figure 4:**
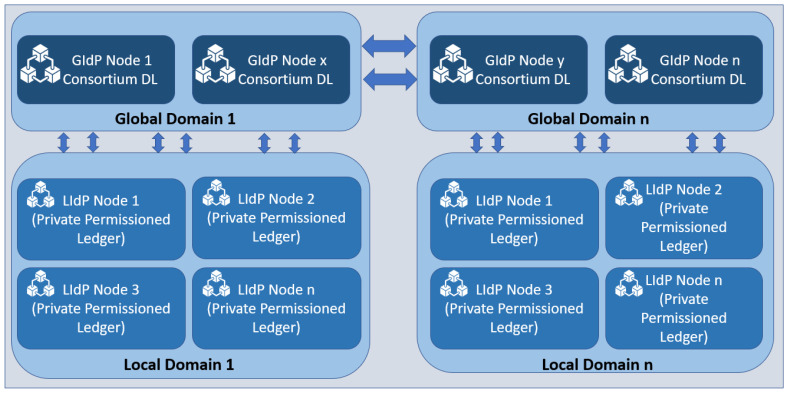
IdP nodes represented as groups.

As our proposed model is fog centric, all the devices are registered with the nearest DLT-based local identity management node (as shown in Figure 3 and Figure 4). The proposed Fog layer DLT-based LIdP network consists of permissioned private blocks where access to information is restricted within a group of entities within a specific location or organization. On the other hand, the cloud layer GIdP network is built upon a consortium network and is shared between different parties. Here, a party can be a group of people within various organizations or devices involved in managing and processing identity information for the devices registered with a LIdP. Edge gateways also keep the identity information of the connected devices. IoT devices only keep track of their identity and the identity information of the gateway it is connected to.

In Figure 4, two groups of LIdPs and GIdPs are presented. However, there could be n number of groups. So, we have mentioned the groups on the right side with the number n. There could be several LIdPs within a particular location, and they collaborate, as shown above. The edge gateways and IoT devices get unique identities assigned and stored in the distributed ledger at the LIdP. The local identity managers will have a private distributed ledger that will collaborate with other fog layer distributed ledgers owned by the same service providers. The GIdPs will be implemented with a consortium distributed ledger to allow collaboration between different identity service providers. There will be no collaboration between the fog layer identity providers if they are from different service providers or at different locations. However, fog layer local identity providers from the same provider will collaborate with each other as they are built upon a private distributed ledger, which enhances the security and privacy of the identity information at the fog layer.

**Figure 5:**
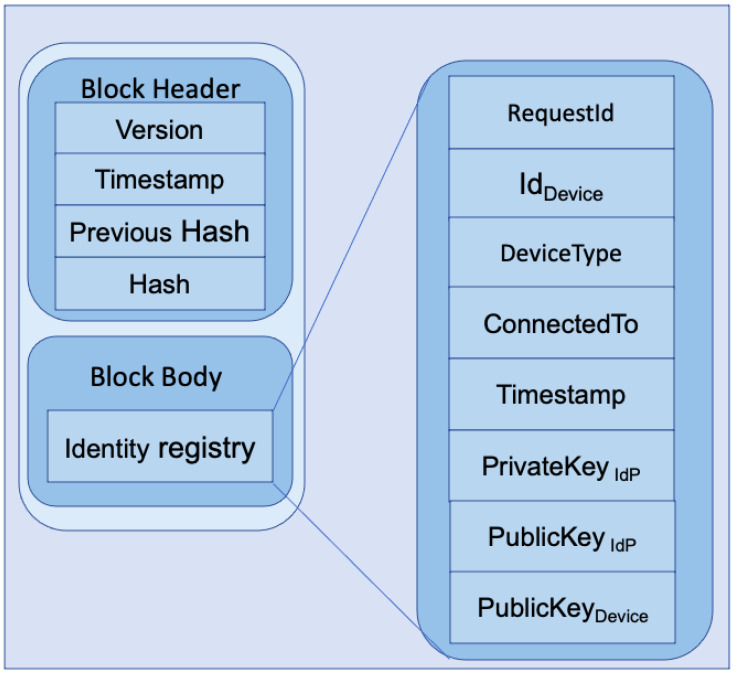
Internal architecture of a single block.

**Figure 6:**
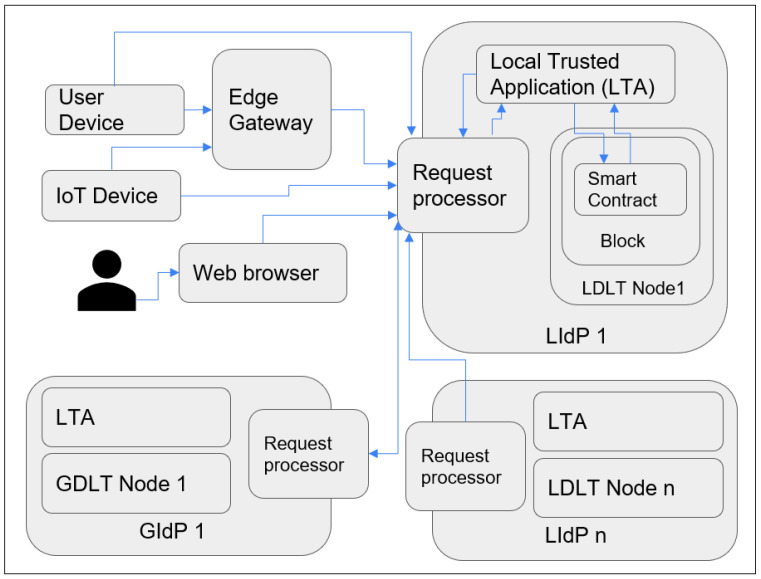
Overall workflow of the system.

In Figure 5, we present the internal architecture of a single block. The left side of the figure shows a single-block architecture. A block consists of a block header and a block body. A block header holds the block’s version, timestamp, previous block’s hash, and current block’s hash. The block body consists of an identity registry where identification information of different devices is stored. A single entry within the identity registry is shown on the right side of the figure, which includes requestId, identity information of IoT device/user device/IoT gateway represented as Id_Device_, device type as DeviceType, device connection information as ConnectedTo, timestamp of request as Timestamp, own private and public key as PrivateKey_IdP_ and PublicKey_IdP_, and public key of the device as PublicKey_Device_. These keys are only used for communication between the IdP and the respective device, which could be an IoT device/user device/IoT gateway. The reason behind saving one’s own public and private key is to communicate uniquely with each device. The private key will stay secure, as access to the ledger should be performed via the Local Trusted Application (LTA) presented in Figure 6. For further details of secure communication protocol, readers are suggested to look at our previous work [20], as the current model is built on top of our previous work. A LIdP shares the identity information of a device with a GIdP if it is requested. In that case, the key information is not shared with the GIdP. Besides the identity of the device, the identity, and the public key of the LIdP are shared so that the GIdP can share it with the requesting LIdP that has got a request from a gateway where the IoT device is moved. The requesting LIdP requests a sync with the other LIdP to sync the actual identity information of the device. The below section describes the workflow of our proposed system.

### 4.4. Workflow of the System

There are several different workflows for our proposed system model. As we have discussed earlier, our proposed model handles different scenarios. We have considered six different workflows for our proposed system to generalize all the scenarios.

Figure 6 represents the overall workflow of the proposed system. The request processor module processes each valid external request before forwarding it to the Local Trusted Application (LTA) module. The LTA module is responsible for further verification of the request and comparison for the data saved in the smart contract or adding a new entry to the local temporary storage for further adding it to the blockchain. As shown in the figure, each device or IdP communicates with another IdP via the request processor. Here the request processor plays an essential role in stopping unnecessary traffic toward LTA.

**Figure 7:**
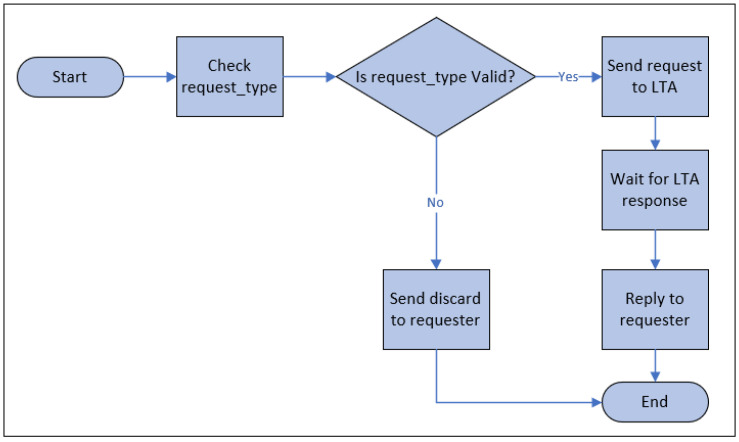
Operation flowchart of Request processor.

Figure 7 represents the flowchart of operations of a request processor. The request processor checks the validity with respect to six different workflows: registration, authentication, authorization, re-registration, revocation, and synchronization. These six types of requests are only known to the community of IdP. The request processor discards all other types of requests. The request processor only knows the six types of requests and verifies them before forwarding to the LTA. For security reasons, the request processor does not verify the identity of the requester. Before forwarding to LTA, the request processor adds tags to the request with three different types: administration, verification, and synchronization. Below, we describe each of the workflows.

#### 4.4.1. Registration

**Figure 8:**
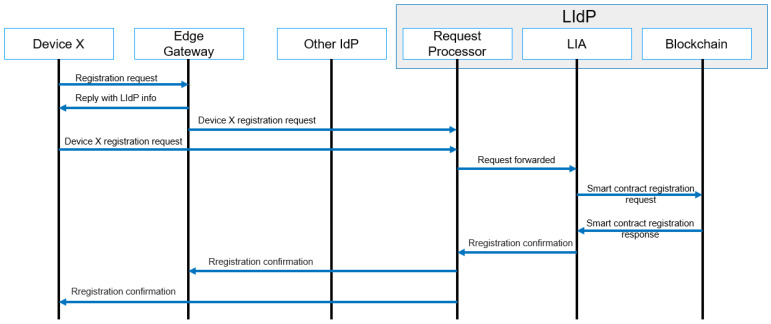
Registration process message flow.

Any user, user device, IoT device, or edge gateway connected to any specific edge gateway needs to be registered with the nearest LIdP. Registration is only performed with the first-time allocation. A generalized message flow of the registration process is given in Figure 8. Here at the first stage, the device type is identified, and based on the device type, and the registration process is carried out. To avoid the complexity of our proposed system, we have not discussed the details of a user registration process in the workflow diagram.

#### 4.4.2. Authentication

**Figure 9:**
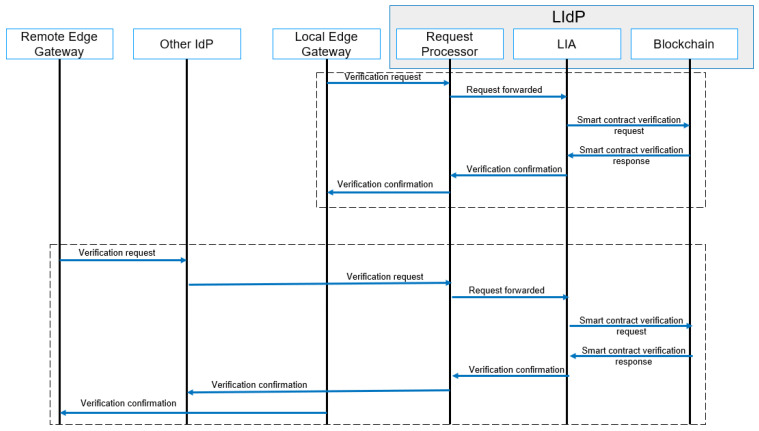
Authentication/Authorization process message flow.

Any user, user device, or IoT device needs to be authenticated before performing any task within the IoT infrastructure. Here, a task can be defined as data transfer, data store, and data access to any other device connected to the specific network. A simplified message flow model for authentication is shown in Figure 9. The same figure applies for both authentication and authorization. The authentication varies based on the type of device. Based on use cases, the IoT devices need to store and transfer data with the nearest edge IoT gateways. The authentications of IoTDs are automated via the EIoTG. Every packet sent from an IoTD to EIoTG consists of unique signatures of the sender device. The EIoTG verifies the signatures by using the public key of the IoTD. The LIdP shares the public key of the connected IoTD. If an IoTD is not authenticated, it is marked as compromised in the EIoTG database for connected devices. At the same time, a notification is sent to the LIdP so that the same device cannot get registered with another EIoTG and performs malicious activities. If required, automated identity verification is performed by the LIdP as it holds the smart contracts, which contain the details about the connected IoTDs with different edge IoT gateways.

#### 4.4.3. Authorization

Authorization is the next step to authentication. Any authenticated user, user device, or IoT device needs to be authorized before performing any task within the IoT infrastructure. Here, a task can be defined as data transfer, data store, and data access to any other device connected to the specific network, such as authentication. A simplified message flow diagram for authorization is shown in Figure 9. The main difference here is to verify the access right of the specific entity that requests access to any resource to perform any task. A few more use cases where authorization is required are data access to an IoT device from a user device, control of a device, device maintenance such as Firmware updates, etc.

#### 4.4.4. Re-Registration

**Figure 10:**
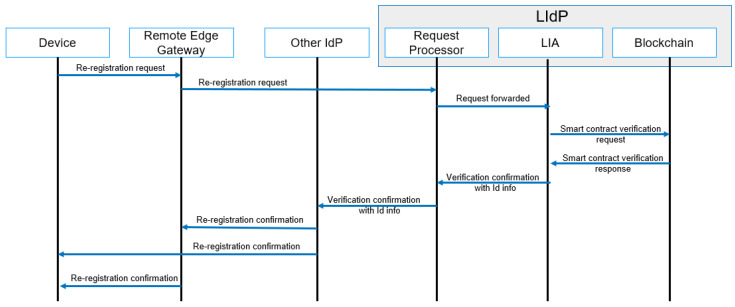
Re-registration process message flow.

A re-registration of a device is performed with the re-location of any IoT device. A device with mobility capability may move to a location where it needs to access resources and complete tasks within a new edge IoT gateway. A simplified message flow diagram for re-registration is shown in Figure 10, where reallocation to a new location based on the device’s movement is performed with re-registration. A re-registration is only done for a device already part of a specific IoT domain and must verify identity to a new edge IoT gateway. Any new device registration is considered under the registration workflow.

#### 4.4.5. Revocation

**Figure 11:**
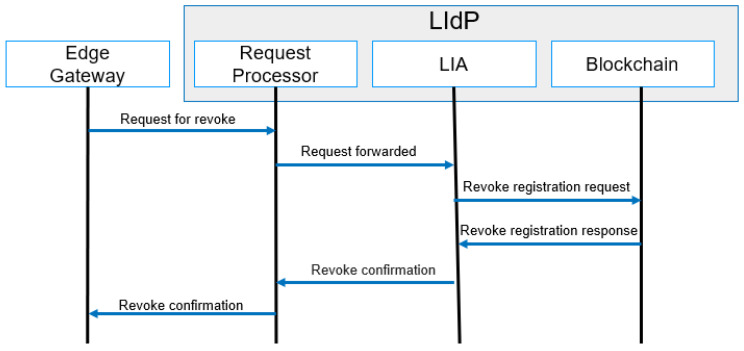
Revocation process message flow.

The revocation of identity is performed for a compromised device. A compromised device is defined as untrusted within the IoT domain due to malicious activity. A simplified message flow diagram for revocation is shown in Figure 11. We proposed a dynamic trust model [71] for trust management for IoT. The same model can be used for identifying untrusted devices, and the identity of untrusted devices can be revoked. Revocation is applicable for both authentication and authorization.

#### 4.4.6. Synchronization

**Figure 12:**
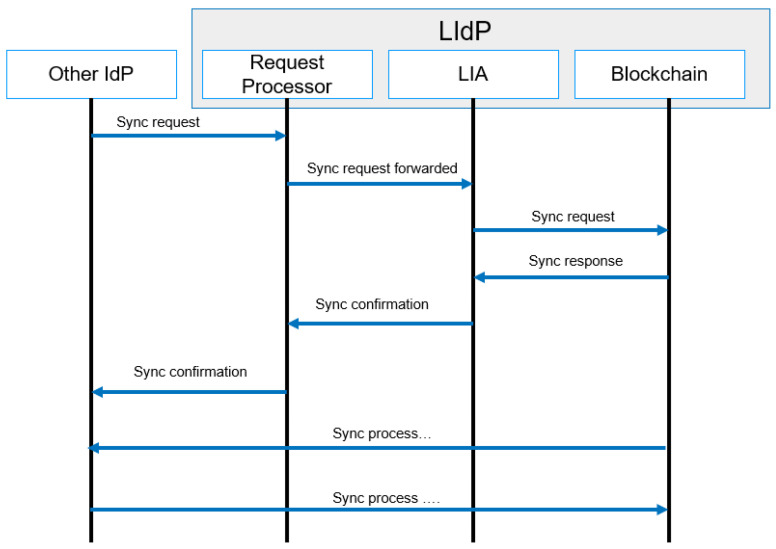
Synchronization process message flow.

The synchronization request always comes from another LIdP or from a GIdP, as we have proposed a DLT-based model. The Ledgers in different LIdPs need to be synchronized. As mentioned above, in our proposed model, the LIdP contains private distributed ledgers that only completely sync with other LIdP within a specific location. The LIdPs sync periodically to store the smart contacts containing identity information of local devices and gateways at the edge layer and within a particular area. LIdP sync is a complete sync between peers. A sync with GIdP is performed with limited data sharing. Figure 12 represents the message flow diagram of the synchronization process. In the next section, we discuss different smart contracts and proposed algorithms for our proposed model.

### 4.5. Smart Contacts and Proposed Algorithms

As previously mentioned, the proposed model should use permissioned blockchain at both GIdP and LIdP. The LIdPs should have private ledgers, and the GIdPs should have consortium ledgers. The LIA module in each IdP performs the primary role here. A total of three main types of smart contacts will be stored in DLT. The smart contacts are designed considering the workflows. Below, we have described each of them. As mentioned earlier, the request processor adds tags for different valid requests before forwarding it to LTA. The LTA first sort requests based on the tags. Algorithm 1 shows the tag identification steps.
**Algorithm 1:** Request Tag extraction**Input:** RequestTag, Request1: **if** RequestTag is IdentityAdministration:2:   **forwared** Request to Algoritm 2;3: **else if** RequestTag is IdentityVerification:4:   **forwared** Request to Algoritm 3;5: **else if** RequestTag is Syncronization:6:   **forwared** Request to Algoritm 4;

#### 4.5.1. Smart Contracts for Identity Administration

Identity administration includes registration, revocation, and re-registration of devices. Each new device must be registered in the distributed ledger with a smart contract. The local identity provider is the initial registration place for a new device. Every new device identity is registered as an entry in the DLT as a smart contract. When a device is compromised, the details of that specific device are also written in DLT with an entry as revoked. An IoT device should have mobility, and the device’s identity should be transferable between nodes. In case a device moves to a new gateway that is not within the range of the current location, new registration of the device is required. This is done by the global identity node, where the device identity is synced in the LIdP at the new location and is recorded in DLT as re-registered. Algorithm 2 presents LTA’s steps for handling registration, revocation, and re-registration requests.
**Algorithm 2:** Identity administration**Input:** RequestType, RequesterId, RequestBody **Output:** ResponseType, ResponseBody1: **if** RequesterId in SmartContact: //RequesterId is only EIoTG and/or Device2:   **if** RequestType is registration: //RequestType registration3:    **if** all the necessary details are present in requestBody:4:      **store** new SmartContact for new device;5:      **return** registrationConfirmation;6:    **else**:7:      **return** RequestNotCompleteError;8:   **else if** RequestType is revocation: //RequestType revocation9:    **if** all the necessary details are present in requestBody:10:     **if** requestedDevice is compromised: //verify device status with peers11:       **store** new SmartContact with revocation details;12:        **return** revocationConfirmation;13:      **else**:14:       **return** RequestMoreEvidence;15:    **else**:16:      **return** RequestNotCompleteError;17:   **else if** RequestType is re-registration: //RequestType re-registration18:     **if** all the necessary details are present in requestBody:19:       **if** peers confirm the previous registration:20:        **store** new SmartContact for re-registration info;21:        **return** re-registrationConfirmation;22:       **else:**23:        **return** re-registrationDecline;24:     **else**:25:       **return** RequestNotCompleteError;26: **else**:27:  **return** RequesterIdNotFoundError;

#### 4.5.2. Smart Contracts for Identity Verification

Identity verification smart contracts are made for the authentication and authorization of devices and edge gateways. After receiving a request for authentication or authorization type, the LTA interacts with the smart contract saved in the DLT to verify the identity or to check the authorization rights. Algorithm 3 presents simplified steps of identity verification.
**Algorithm 3:** Identity verification**Input:** RequestType, RequesterId, RequestBody **Output:** ResponseType, ResponseBody1: **if** RequesterId in SmartContact: //RequesterId is only EIoTG or LIdP2:   **if** RequestType is authentication: //RequestType authentication3:    **if** all the necessary details are present in requestBody:4:      **verify** identity with details in SmartContact;5:      **return** AuthenticationConfirmation; // Can be True or False6:    **else**:7:      **return** RequestNotCompleteError;8:   **else if** RequestType is authorization: //RequestType authorization9:    **if** all the necessary details are present in requestBody:10:     **verify** ACL details in SmartContact;11:     **return** AuthorizationConfirmation; //Can be True or False12:   **else**:13:     **return** RequestNotCompleteError;14: **else**:15:  **return** RequesterIdNotFoundError;

#### 4.5.3. Smart Contracts for Identity Synchronization

The identity synchronization smart contacts are for peer-to-peer communication requests for the synchronization of different DLTs. Our proposed model is based on private and consortium ledgers, so the synchronization requests are significant. Only allowed nodes are synced between each other, and all other requests related to sync are dropped at the LTA. The identity synchronization is performed between LIdP-LIdP and LIdP-GIdP.

**Figure 13:**
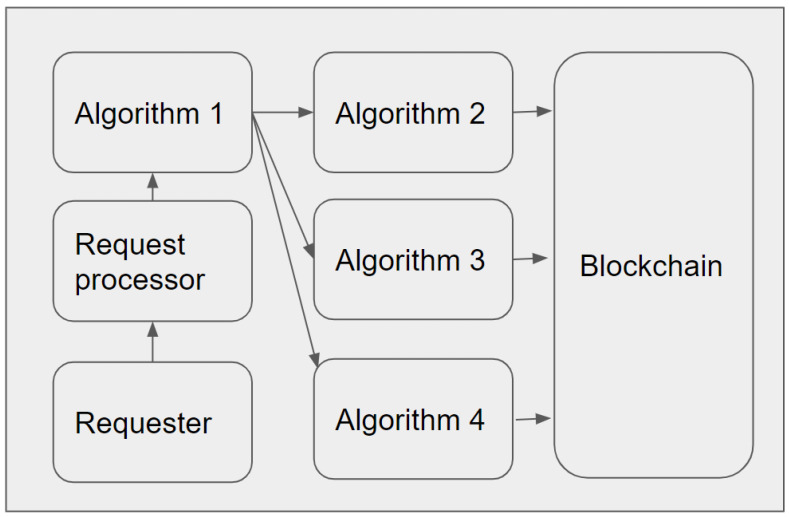
Computation logic of DIdM-EIoTD in a LIdM.

Algorithm 4 represents the steps taken by the LTA for sync requests. LTA checks entries for requesters in smart contracts stored in DTL. Only trusted and authenticated LIdP and GIdP get authorization for synchronization. Furthermore, the sync with GIdP is limited and all the details are not synced with the GIdP. On the other hand, LIdP which is located within certain location and considered as within local network are allowed for full sync.
**Algorithm 4:** Identity syncronization**Input:** RequestType, RequesterId, RequestBody **Output:** ResponseType, ResponseBody1: **if** RequesterId in SmartContact: //RequesterId is only LIdP or GIdP2:   **if** RequestBody is complete: //RequestBody verification3:    **if** requester is LIdP:4:       **if** requester is in LocalNetwork:5:         **return** SyncronizationAllowedConfirmation (FullSync);6:       **else**:7:         **return** UnauthorizedSyncronizationRequest;8:     **else if** requester is GIdP:9:      **if** GIdP is in peer:10:       **return** SyncronizationAllowedConfirmation (LimitedSync);11:      **else:**12:       **return** UnauthorizedSyncronizationRequest;13:    **else:**14:       **return** InvalidRequesterError;15:   **else**:16:     **return** RequestNotCompleteError;17: **else**:18:  **return** RequesterIdNotFoundError;

Figure 13 presents the computation logic of our proposed model DIdM-EIoTD in an LIdM, where the connections between algorithms and the DLT are shown. The next section presents the results of simulations and related analyses for our proposed model.

## 5. Results and Analysis

This section presents the simulation results of our proposed model. We have divided this section into three different sub-sections. First, in Section 5.1, we introduce a formal security analysis and description of the Scyther simulation experimental results. After that, in Section 5.2, we present the results of the model-checking tool SPIN. At the end, in Section 5.3, we present the results of FobSim simulation.

### 5.1. Formal Security Analysis

**Figure 14:**
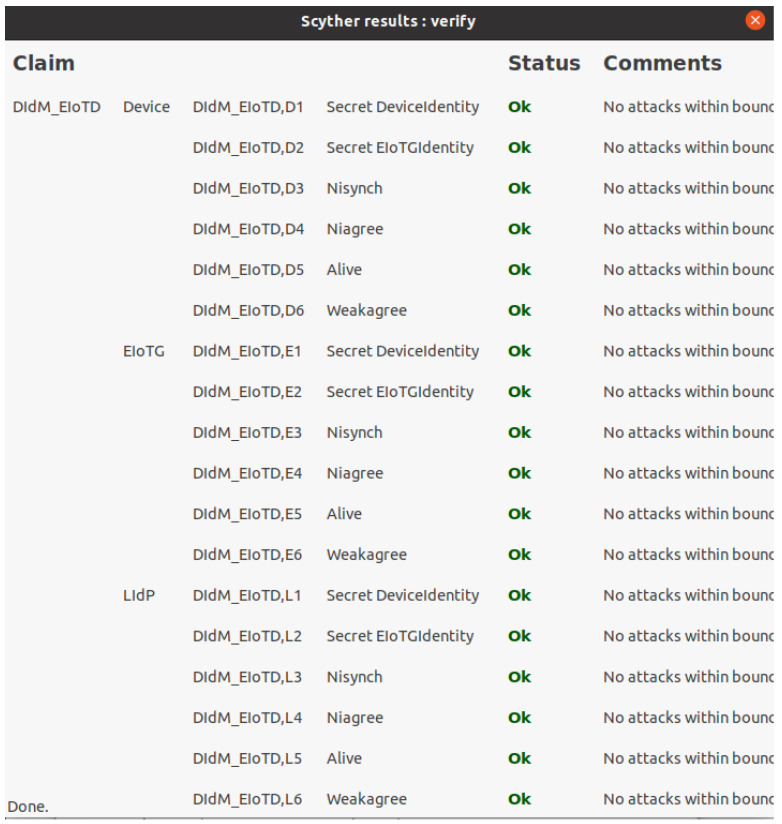
Scyther verification results for interaction between Device, EIoTG and LIdP.

We have used Scyther [71] security verification tool for the security analysis of our proposed model. Scyther tool allows claims of different security properties: Secret, Nisynch, Niagree, Alive, Weakagree, etc. Figure 14 presents verification results for the secret of Device identity as well as Edge IoT Gateway identity as well as Nisynch, Niagree, Alive, and Weakagree. As described above, our proposed model will allow different types of device identity management, but for simplicity, we have only presented everything as a device during verification. The Scyther simulation was done in Intel (R) Core (™) i7-6500 CPU @ 2.50 GHz 2.59 GHz and 16.0 GB internal memory (RAM) with Ubuntu 20.04 operating system.

**Figure 15:**
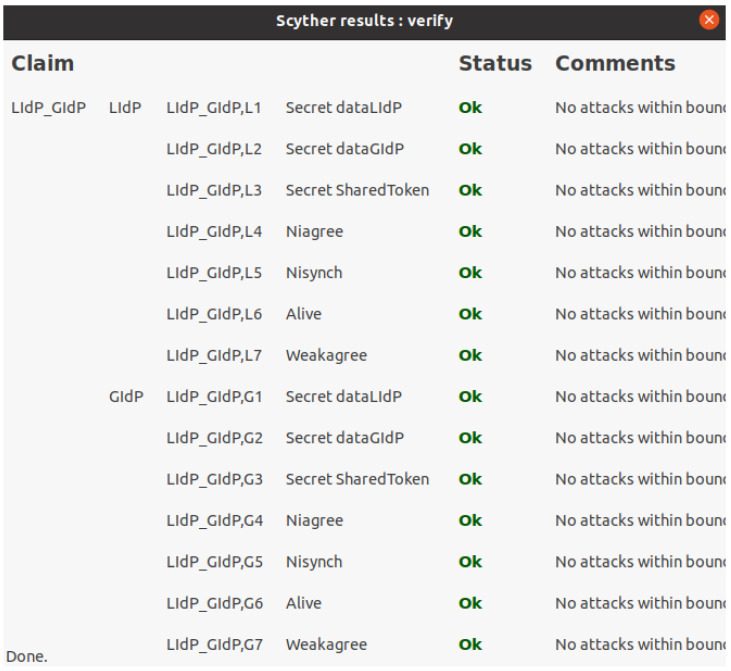
Scyther verification results for interaction between LIdP and GIdP.

As mentioned earlier, the GIdP and LIdP will sync with each other. Furthermore, the LIdPs within specific locations will collaborate as those will be within a private permissioned distribution ledger. Figure 15 presents the security verification for interaction between a GIdP and a LIdP, which verifies the secret of data transferred between each other and other standard security properties Nisynch, Niagree, Alive, and Weakagree.

### 5.2. SPIN Model Checker Results

SPIN is a popular model checker that verifies the states of different complex systems [72,73]. We have used Spin Version 6.4.9 to simulate our proposed model. The simulation was conducted using an Intel (R) Core (™) i7-6500 CPU @ 2.50 GHz 2.59 GHz and 16.0 GB internal memory (RAM) with Ubuntu 20.04 operating system. Our proposed model was verified per the scenario presented in Figure 3 and Figure 5. Below, we have the verification results of the scenario of Figure 3, which is an interaction between different IdP. Table 2 represents the SPIN verification result for interaction between different IdP.

From the above, we can see that the simulation time increased a bit with extra options. Table 3 represents the complexity of increasing status with respect to depth and number of states for normal SPIN search, search with acceptance cycle as well as search with safety. We only noticed a slight difference in simulation time of around 0.10 s.

**Figure 16:**
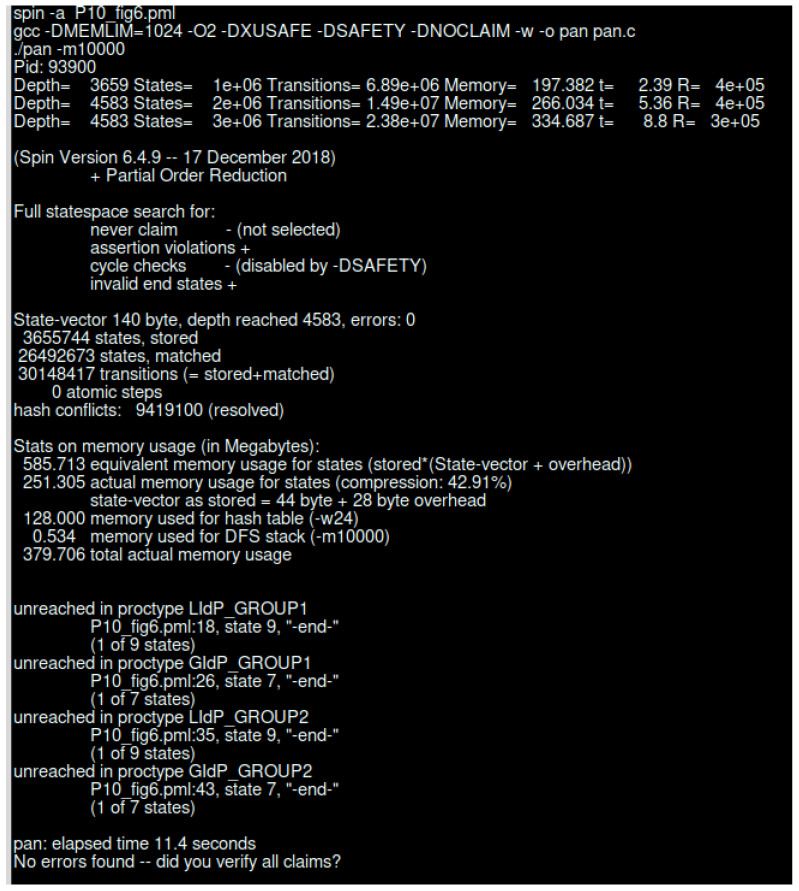
SPIN verification result for LIdP and GIdP interaction.

Figure 16 represents the SPIN verification results without any error state. As we have performed a simulation for the continuous running of the system, the end state is unreachable. We have created four different groups of IdP where two groups represent the GIdP, and two other groups present the LIdP.

Table 4 represents the compression effect of the simulation. The SPIN simulator allows different types of compression during the verification. We have considered a standard run of the simulation followed by normal compression, hash compact compression, bitstate compression, bitstate compression with optimization, and bitstate compression with different array sizes and optimization.

**Figure 17:**
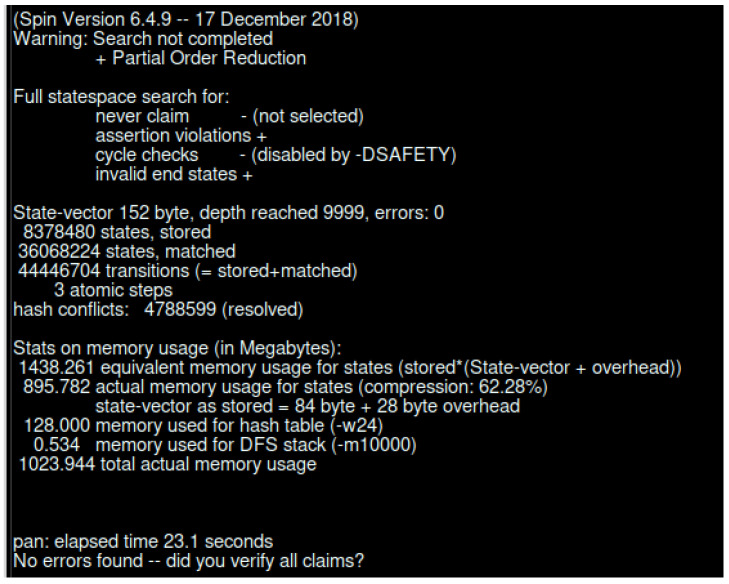
SPIN verification for interaction between LIdP and different devices.

In Figure 17, we present the verification result for the scenario in Figure 5. The verification took 23.1 s, and produced 44,446,704 transitions. The total amount of memory usage was 1,023.944 Mb.

### 5.3. FobSim Simulation Results

We used FobSim [74] to analyze the behavior of our fog-based identity management solution with DLT deployment at the fog layer and at the edge/user device layer. The simulation was run in an Intel (R) Core (™) i5-6200U CPU @ 2.30 GHz 2.40 GHz with 16.0 GB internal memory (RAM) and Windows 10 Professional operating system. FobSim is an open-source simulator that allows deployment of Blockchain/DLT at the fog layer and used device layer. FobSim allows simulation with Proof-of-Work (PoW), Proof-of-Stake (PoS), Proof-of-Authority (PoA), Proof-of-Elapsed-Time (PoET), and delegated Proof-of-Stack (dPoS) as a consensus mechanism. We used Proof-of-Authority (PoA) as it is best suits our simulation needs. The simulator supports payment/Trading, data management, identity management, and computational services through smart contracts. We used the identity management service for our simulation. The authors of FobSim mention miners which is verified in our scenario. A verifier can only verify a block based on its Proof-of-Authority (PoA).

**Figure 18:**
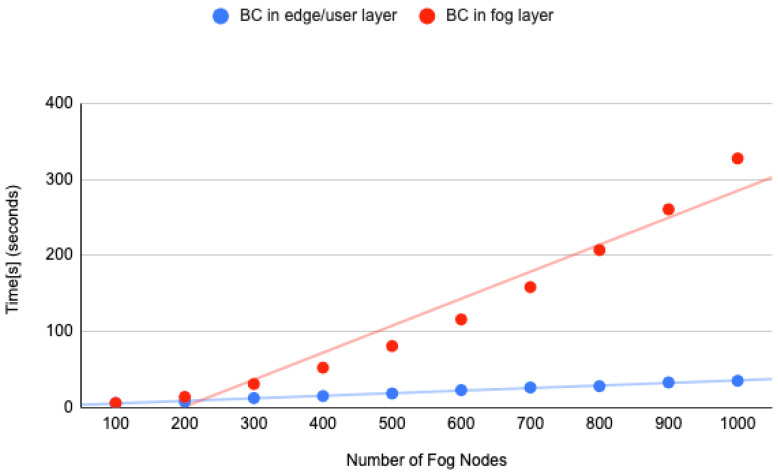
FobSim simulation result.

We have performed the simulation with the number of fog nodes from 100 to 1000, as presented in Figure 18. The simulation parameter configuration details are shown in Table 5. The Blockchain was placed at the Fog layer and the user device layer. As we can see in the graph as well as in Table 6, the simulation took more than 300 s when we used 1000 fog nodes, and the Blockchain was deployed at the fog layer. On the other hand, the simulation with blockchain deployment at the user device/edge layer took less than 20 s. A cloud layer deployment of Blockchain will be worse than the deployment at the fog layer as it is far from the device layer.

## 6. Discussion

As described in [49], scalability, interoperability, and mobility are three major concerns that need to be addressed using traditional centralized identity management systems. In our proposed identity management solutions, we have considered all of these. Our recommended identity management solution will allow scalability by combining permissioned and consortium distributed ledger technologies. At the edge layer, the permissioned ledgers will collaborate locally and share identity data with the cloud layer ledgers, where we have recommended a consortium-based DLT approach. This will enhance scalability as we are allowing cloud storage of identity information. It will enable interoperability, as we suggest a consortium-based distributed ledger approach. Finally, it will enable the mobility of devices as local and global identity information is shared between the locally configured distributed ledger and globally configured distributed ledger as per demand from the edge gateways.

Other significant concerns identified by the authors in [49] are security, privacy, trust, and user-centric approaches. We have proposed a fog-computing-based distributed, decentralized identity management solution for IoT devices. The proposed system has the following benefits:

- Distributed identity management for edge IoT devices;
- Decentralized identity verification solution at the fog layer of the IoT paradigm;
- End-user data privacy using permissioned Blockchain;
- Digital identity for user devices as well as end-users;
- End-to-end data security using a secure communication protocol;
- Trustworthy collaboration between devices.

The main reason for using permissioned smart contracts and permissioned Blockchains is to enhance user data privacy at the edge layer of IoT. Users’ personal information are saved locally at the edge layer/fog layer of the IoT paradigm and only accessible within the respective parties, which increases security and ensures privacy. Our proposed approach is decentralized and distributed. Identity management is possible locally, which allows quick responses for quick decision-making at the edge IoT layer without verification from the cloud layer.

## 7. Conclusions

To bring the identity service towards the edge, we have proposed distributed ledger-based identity providers at the fog layer of the IoT paradigm. The proposed model can be adapted for identity management of users, handheld devices, edge gateways, and IoT devices assigned for specific tasks. As mentioned earlier herein, the reason for using a permissioned ledger is that we aim to enhance security and privacy in combination with identity management. Although we have verified our proposed model with several simulation tools, we have not deployed the model in real-life scenarios with practical IoT devices and DLTs, which presents a limitation of our work. Future works should address the practical implementation of our proposed model in real-life situations. Furthermore, the proposed model will be integrated with our proposed multi-agent-based edge gateway solution [75] for trust management and machine-learning-based privacy solution model [15] for complete security, trust, and privacy solution for the IoT paradigm.

## Figures and Tables

**Table 1:** More consensus mechanisms.

Consensus Mechanism	Reference
Proof of Deposit (PoD)	[35]
Yet Another Consensus (YAC)	[35]
Practical Byzantine Fault Tolerance (PBFT)	[35,36,37,38]
Delegated Byzantine Fault Tolerance	[37]
Federated Byzantine agreement	[35,36]
Proof of Participation and Fee	[37]
Proof of Search	[37]
Proof of Contribution	[37]
Proof of Stake Velocity (PoSV)	[37,38]
Proof of Exercise	[37]
Proof of Learning	[37]
Proof of Space (PoSpace)	[37,38]
Proof of Vote (PoV)	[37]
Proof of TEE-Stake (PoTS)	[37]
Proof of Reputation (PoR)	[35,38,49]
Proof of Reputation-X	[37]
Delegated Proof of Reputation (DPoR)	[37]
Proof of Property	[37]
Proof of Block and Trade	[37]
FastBFT	[37]
Proof of QoS (PoQ)	[37]
Proof of Validation	[38]
Hashgraph	[38]

**Table 2:** SPIN verification result for interaction between different IdP.

Verification Details	State-Vector (Byte)	Depth Reached	Transitions	Time (s)	Memory (Mb)	Rate (States/s)
Normal search	140	4583	30,148,417	10.1	379.706	361,954.85
Search with acceptance cycle	140	4583	30,148,417	10.8	379.706	337,869.13
Search with safety	140	4583	30,148,417	11.2	379.706	326,989.62

**Table 3:** Complexity representation of SPIN simulation.

Depth	No of States	Transitions	Memory (Mb)	Time (s)	Rate (States/s)
3659	1 × 10^6^	6.89 × 10^6^	197.382	2.07	5 × 10^5^
4583	2 × 10^6^	1.49 × 10^7^	266.034	4.72	4 × 10^5^
4583	3 × 10^6^	2.38 × 10^7^	334.687	7.8	4 × 10^5^

**Table 4:** Compression effect on the simulation results.

Type of Run	Rate (States/s)	Memory (Mb)	Time (s)
Standard	352,871.04	379.706	10.4
Normal Compression	157,915.51	323.944	23.1
Hash compact compression	497,625.48	267.987	7.34
Bitstate compression	359,067.49	17.413	10.2
Bitstate compression with optimized search	367,367.54	17.413	9.95
Bitstate with array size 2^30^ array size with optimization	318,167.36	129.413	11.5
Bitstate with array size 2^34^ array size with optimization	252,294.27	2049.413	14.5

**Table 5:** FobSim simulation parameter configuration.

Simulation Parameter	Value
No. of fog nodes	100–1000
No. of devices per fog node	100
No. of task per device	5
No. of miners/EIoTG	100
No. of neighbors per miners/EIoTG	3
No. of task per block	5
Gossip activated	True
Asymmetric key length	512
Delay between fog neighbors	12 ms
Delay between end-user/device neighbors	1000 ms

**Table 6:** FobSim simulation results with BC deployment at edge/user layer and fog layer.

Number of Fog Nodes	BC in Edge/User Layer	BC in Fog Layer
100	6.05780482292175	6.22806930541992
200	8.13564014434814	14.2369947433471
300	12.6292879581451	31.1524369716644
400	15.3436908721923	52.6077961921691
500	18.693144083023	80.9993338584899
600	23.1540505886077	116.00711941719
700	26.5926034450531	158.398232460021
800	28.3022751808166	207.182087421417
900	33.2225153446197	260.620384931564
1000	35.4516329765319	327.477404356002

## Data Availability

Not applicable.

## Appendix A

**Table A1:** Abbreviations and descriptions of different terms.

Abbreviations	Description
IoT	Internet of Things
CA	Consensus Algorithm
SC	Smart Contracts
SP	Service Provider
DL	Distributed Ledger
TTP	Trusted Third Party
IoTD	Internet of Things Device
EIoTG	Edge Internet of Things Gateway
DLT	Distributed Ledger Technology
BC	Blockchain
FG	Fog Gateway
FC	Fog Computing
P2P	Peer-to-Peer
IdP	Identity Provider
LIdP	Local Identity Provider
LTA	Local Trusted Application
GIdP	Global Identity Provider
LBCN	Local Blockchain Network
GBCN	Global Blockchain Network
Id_U_	User Identity
Id_Ud_	User Device Identity
Id_IoTD_	Identity of Internet of Things Device
Id_EIoTG_	Identity of Edge Internet of Things Gateway
Id_LIdP_	Identity of Local Identity Provider
Id_GIdP_	Identity of Global Identity Provider
